# Multimodal Stepped Care Approach Involving Topical Analgesics for Severe Intractable Neuropathic Pain in CRPS Type 1: A Case Report

**DOI:** 10.1155/2011/319750

**Published:** 2011-10-17

**Authors:** David J. Kopsky, Jan M. Keppel Hesselink

**Affiliations:** ^1^Institute for Neuropathic Pain, Vespuccistraat 64-III, 1056 SN Amsterdam, The Netherlands; ^2^Institute for Neuropathic Pain, Bosch en Duin, The Netherlands

## Abstract

A multimodal stepped care approach has been successfully applied to a patient with complex regional pain syndrome type 1 and severe intractable pain, not responding to regular neuropathic pain medication. The choice to administer drugs in creams was made because of the intolerable adverse effects to oral medication. With this method, peak-dose adverse effects did not occur. The multimodal stepped care approach resulted in considerable and clinically relevant decrease in pain after every step, using topical amitriptyline, ketamine, and dimethylsulphoxide.

## 1. Introduction

Complex regional pain syndrome (CRPS) is a challenging pain syndrome usually starting after a trauma or surgery. Two types of CRPS are distinguished: CRPS type 1 (CRPS-1) without demonstrable nerve lesions and related to a trauma and CRPS type 2 based on objective nerve damage, most commonly caused by severe trauma. CRPS-1 is recently recognized as a chronic neuropathic pain syndrome and typically develops in an extremity after tissue trauma [1]. CRPS type 2 is formerly known as causalgia, while CRPS-1 is formerly known as Sudeck's atrophy, algodystrophy, posttraumatic dystrophy, and reflex sympathetic dystrophy. Besides the neuropathic pain symptoms, such as burning pain and allodynia, CRPS is associated with autonomic, trophic, and motor function changes. Consequently, the following symptoms in the affected region can occur: edema, altered sweating, skin color, and skin temperature, changes to the skin, hair, and nails, loss of strength, decreased active range of motion, and tremor. The estimated incidence is 5.46 to 26.2 new cases per 100,000 annually [2, 3]. The current understanding of the pathophysiology of CRPS is that multiple mechanisms are involved, such as reduced density of C- and A*δ*-fibers, central and peripheral sensitization, altered vasomotor function, changes in catecholamine concentration and its receptors, increased inflammatory and decreased anti-inflammatory factors, decreased representation on the somatosensory cortex, genetic predisposition, and psychological factors [1]. 

Many therapies have been evaluated, though no medical treatment has shown robust efficacy [1, 4]. Interdisciplinary approach for functional restoration, pain management, and psychological support can enhance the chance of full recovery [1, 5]. This is clearly an important issue, as incomplete resolution of signs and symptoms is common and only one-third of the patients reach full recovery [6]. Therefore multimodal therapy, an emerging method in the treatment of neuropathic pain [7, 8], is also explored for treating intractable pain in CRPS patients.

This paper details successful treatment of a CRPS-1 patient with severe intractable neuropathic pain, following a multimodal stepped care approach, using creams of amitriptyline, ketamine, and dimethylsulphoxide (DMSO).

## 2. Case Report

A 42-year-old Caucasian male was referred to our Institute for Neuropathic Pain with complaints of severe intractable pain around the left ankle. Progressive pain developed in November 2007, two months after a fall from the stairs. The immediate symptoms following the fall were a massive edema and hematoma of the left leg, with signs and symptoms of an ankle distortion. Subsequently he was diagnosed with CRPS-1. At the time of diagnosis the left ankle was colder than the surrounding tissue, and trophic changes were apparent. Symptoms of neuropathic pain, such as pricking, tingling, shooting pain, hyperalgesia, and allodynia, were present. MRI scans, X-rays, lumbar punction, and laboratory blood tests did not reveal any pathology, thus supporting the diagnosis of CRPS-1. Nerve conduction studies revealed a slight distal motor latency of the nervus peronaeus superficialis, but no significant difference was observed between the left nerve and the right nerve.

Analgesics such as gabapentin, pregabalin, tramadol, and NSAIDs (nonsteroidal anti-inflammatory drugs), all given in the highest dosages, were not effective. The patient experienced only adverse effects, such as drowsiness and feeling like a zombie. As a matter of fact, high dose of tramadol resulted in hospitalization due to an opiate-induced ileus. Only amitriptyline 25 mg orally was somewhat effective in pain reduction, however the adverse effects limited its further use.

On the 19th of May 2010, at his first visit to our institute, he complained of severe pain. The pain score was 9 out of 10 on the 11-point numerical rating scale (NRS), where 0 is total absence of pain and 10 is the worst pain imaginable. On the short-form McGill Pain Questionnaire (SF-MPQ), he scored 26 out of 45 with the following severe pain symptoms: stabbing, sharp, tender, tiring-exhausting, and fearful. On the Neuropathic Pain Symptom Inventory (NPSI), he scored 44 out of 100 (within this scale his subscores on the NRS were squeezing 7, pressure 7, stabbing 8, tingling 8, provocation of increase of pain by pressure 7, and provocation by something cold 7). According to the part of the Brief Pain Inventory (BPI) concerning interference of pain with the patient's life, the pain interfered profoundly with daily activities as shown in Table 1.

Neurological examination was normal, apart from allodynia after the tuning fork test on the left maleolus lateralis, as well as after light rubbing on the painful area. We decided to prescribe amitriptyline 5% cream three times daily, because the pain had components of neuropathic pain, such as allodynia, hyperalgesia, tingling, and stabbing. Previously, on oral amitriptyline the patient experienced some relief; however, he also experienced dose-limiting adverse effects, most probably peak-dose effects. Therefore, we decided to apply amitriptyline topically, because amitriptyline cream acts as a slow-release formulation [9]. One month later his overall pain score on the NRS decreased about 30%, from 9 to 6 or 7, without any adverse effects.

Ketamine cream has been found to be effective in CRPS patients in some pilot trials [10, 11], and thus we decided to add topical ketamine 10% cream 3 times daily. After one month the overall pain was further reduced to 5 on the NRS.

The third cream, DMSO 50%, was added because of positive studies on CRPS patients [12–14], and because DMSO acts as a penetration enhancer for topical drugs [15]. One month later the pain decreased further by more than 50%, to 3 on the NRS. During the last evaluation, on 27th of October 2010, he reported that the pain was virtually gone, with the total pain score of 1.5 on the NRS. This pain relief lasted 4 months while using the creams 3 times daily. Thereafter the patient gradually reduced the frequency of application and is applying the 3 creams now only in the morning with sustained alleviation in the last 8 months.

Altogether, in 5 months of treatment; the pain decreased profoundly: from 26 to 6 out of 45 on the SF-MPQ, and from 44 to 12 out of 100 on the NPSI. It must be stressed that his quality of life increased profoundly (Table 1) with no adverse effects during the whole treatment period.

## 3. Discussion

This paper concerns the complexity of treating a patient suffering from CRPS-1. Until now only a few controlled studies on therapies of CRPS have been conducted [16]. Frequently, treatment regimes resulting from various studies of neuropathic pain syndromes have been transferred to CRPS patients [17]. Efficacy of NSAIDs in CRPS patients has not been systematically evaluated so far [4]. However, this class of drugs is often used as the primary therapy, prior to referral to a specialized institution [16]. There is insufficient evidence that opioids and local anesthetics, such as lidocaine and botulinum injections, are effective in treating neuropathic pain in CRPS [4]. On the other hand, there are indications that ketamine, DMSO cream, antidepressants, and anticonvulsants are effective in treating the neuropathic pain in CRPS [4].

Before the patient of this case report was referred to our clinic, several different analgesics have been prescribed and taken, however without success and with considerable adverse effects. Our strategy was to administer topical formulations of evidence-based established analgesics for neuropathic pain with localized activity. It was also aimed at reducing the risk of peak-dose adverse effects, usually seen after taking oral medication [18, 19]. Probably both local and systemic effects resulted in pain reduction. On one hand, no plasma levels of the active compounds were detected after ketamine 10% [10]; on the other hand, adverse effects were reported after amitriptyline 10%, possibly being a poor metabolizer and therefore as a result an increased plasma level [9]. Future research has to clarify this issue. 

Topical strategy proved to be successful in our patient, as no adverse effects were reported. 

The rationale of our multimodal stepped care approach can be explained as follows. As the first step of our treatment schedule we selected topical amitriptyline 5%. It is well known that oral amitriptyline is the first choice in the treatment of neuropathic pain, beause the numbers needed to treat is low: 2 to 3 [20]. This means that 2 or 3 patients have to be treated so that 1 patient has at least 50% pain reduction. It is also documented that the topical administration of amitriptyline is efficacious and safe in dosages of 2% to 4% in various neuropathic pain syndromes and is usually combined with ketamine [21–23]. In previous history our patient responded to some extent to oral amitriptyline; however, adverse effects limited its use. Therefore, topical amitriptyline was well justified.

In the second step we added topical ketamine. Several small trials in CRPS patients supported the analgesic use of ketamine [9, 11, 24–27], although most trials used other forms, such as intravenous application. Ketamine acts on the N-methyl-D-aspartic acid receptor (NMDAR), located on nerve fibers and in the central nervous system [28]. The pain-reducing effect of ketamine cream could be explained, because increased inflammatory factors are present in CRPS, and inflammation in the periphery increases the number of NMDAR on peripheral nerve fibers [29]. Our choice to use topical ketamine 10% is based on a positive double-blind cross-over trial [10].

In the third step we added DMSO 50% cream. The use of DMSO as an analgesic in CRPS patients is well documented [12–14]. Furthermore, DMSO acts as a penetration enhancer for other topically administered drugs, in particular for amitriptyline and ketamine, which we used [15].

Medication as monotherapy is usually not sufficient to treat effectively neuropathic pain, such as in CRPS. Adverse effects can also be the cause of not reaching sufficient analgesic doses. Multimodal therapy is well established in other diseases such as hypertension, asthma and cancer [30–32]. 

Recent insights in treating neuropathic pain clearly indicate the advantages of the multimodal approach targeting different levels of pain signaling pathways [33, 34]. Therefore, such a multimodal stepped care approach can be considered in CRPS patients with severe intractable pain, with the goal of achieving maximal analgesic effects, minimal adverse effects and optimal compliance.

## Figures and Tables

**Table 1:** Interference of pain in patient's life.

	19-5-2010	27-10-2010
General activity	7	1.5
Mood	6	4
Walking	7	1.5
Work	10	1.5
Relations with others	2	0
Sleep	6	0
Enjoy	8	0

0: does not interfere and 10: interferes completely.
